# Burden of falls attributable to low bone mineral density among people aged 60 years and over in China from 1990 to 2019

**DOI:** 10.3389/fpubh.2023.1204497

**Published:** 2023-06-28

**Authors:** Yali Fu, Lei Ba, Nianqing Lü, Huafeng Yang, Xin Hong, Jinyi Zhou, Zhiming Sun

**Affiliations:** ^1^Jiangsu Health Development Research Center, Nanjing, Jiangsu, China; ^2^National Health Commission Key Laboratory of Contraceptives Adverse Reaction Surveillance, Nanjing, Jiangsu, China; ^3^Jiangsu Provincial Medical Key Laboratory of Fertility Protection and Health Technology Assessment, Nanjing, Jiangsu, China; ^4^Nanjing Municipal Center for Disease Control and Prevention, Nanjing, Jiangsu, China; ^5^Jiangsu Provincial Center for Disease Control and Prevention, Nanjing, Jiangsu, China

**Keywords:** falls, low bone mineral density, osteoporosis, age-period-cohort analysis, burden

## Abstract

**Objective:**

Falls in older people have become a major public health, economic and societal problem. Osteoporosis predisposes older adults to high risk of falls, which were the most common outcome attributable to low bone mineral density (LBMD). In this study, we analyze the long-term trends in falls burden attributable to LBMD among people aged 60 years and over from 1990 to 2019, using data from the Global Burden of Diseases, Injuries, and Risk Factors Study 2019 (GBD 2019).

**Methods:**

Data from GBD 2019 were used to assess the long-term trends in mortality and disability-adjusted life-year (DALY) rates by Joinpoint regression. The age-period-cohort (APC) model was used to evaluate the effects of age, period and cohort on mortality rate of falls attributable to LBMD.

**Results:**

The mortality and DALYs rates of falls attributable to LBMD among people aged 60 years and over increased from 1990 to 2019, with average annual percentage changes (AAPCs) of 1.74% (95% CI: −1.47 to 2.01%) and 0.99% (95% CI: 0.80–1.19%), respectively. APC analysis revealed that the mortality rate due to LBMD significantly increased among the older people over the age of 75 years. The risk of falls mortality due to LBMD during the period of 1990–2019 initially declined but later elevated. An overall increasing risk for falls death attributable to LBMD was presented across birth cohorts, but in cohorts born after 1930, the upward trend has slowed down. The overall net drift per year attributable to LBMD was above 0. The corresponding results showed that the negative impact of period and cohort effects among males was more pronounced than those among females.

**Conclusions:**

Falls attributable to LBMD remain an ongoing health burden in the older people in China, and the mortality has been on the rise from 1990 to 2019, especially among the older people aged 80+ years group. The prevention and treatment of LBMD should be emphasized, especially among males and oldest-old people. Furthermore, there is an urgent need to strengthen the implementation of system-wide, integrated and effective public health policies and other health interventions in China.

## Introduction

Globally, falls are the second leading cause of unintentional injury death, with an estimated 684,000 death cases each year, which has become a major public health, economic and societal problem (1). According to the Global Burden of Diseases, Injuries, and Risk Factors Study 2019 (GBD 2019), about 752,537 people worldwide died from falls, of which 73.63% were aged 60 years and over (2). One-third of people aged 65 years and over fall at least once every year (3–6). And they have an increased risk of death or serious injury from falls with advancing age and frailty level (7, 8). Data from China's national statistics showed that about 40 million older adults fell at least once a year in China, and the direct medical cost was more than 5 billion yuan per year (9, 10). In 2019, among people aged 60 years or over in China, 9.76 million severe falls occurred, resulting in a substantial loss of more than 3.18 million disability-adjusted life-years (DALYs) and the number will continue to increase with aging.

It has been well documented that a range of biological, behavioral, socioeconomic, environmental and other risk factors affect falls risk among older adults. For example, LBMD is a state of decreased bone mass with two stages, osteopenia and osteoporosis (11, 12), which was one of the risk factors for falls. A recent survey reported that more than 40 million older adults have been diagnosed osteopenia in the United States and 66% of Australians older than 50 years have osteoporosis (13, 14). Based on data from the first epidemiological survey of osteoporosis in China, the prevalence of osteoporosis was 32.0% in the group over 65 years old, while the prevalence of LBMD was 46.4% in the group over 50 years old (15). China has the largest population in the world, and the prevalence of osteoporosis has also escalated in recent years, with an estimated 212 million people living with osteoporosis in China by 2050.

Möckel (11) found that there was a risk of falls of up to 6% among patients with LBMD and osteoporosis in placebo arms of clinical trials. Immonen et al. (16) found that the clusters where osteoporosis was the defining factors had significantly higher risk of falls (OR = 5.65, 95% CI: 1.23–25.85, *p* = 0.026). Osteoporosis or osteopenia often occurs in combination with sarcopenia, known as Osteosarcopenia. A large population-based prospective cohort study in Chilean showed that osteosarcopenia is associated with increased falls, fractures, functional impairment, and mortality (12).

Several studies have reported the variation of the burden of falls among people over the age of 60 years in China at both national and subnational levels over the past three decades (17). However, few studies have examined the burden of falls attributable to LBMD, especially in terms of temporal trends. Therefore, we aim to comprehensively analyze the long-term trends in falls burden attributable to LBMD in China from 1990 to 2019, including mortality and DALYs. An age-period-cohort model was estimated to analyze the independent effects of age, time period, and birth cohort. Aiming to provide evidence for the prevention and management of health effects of LBMD-related falls in China.

## Materials and methods

### Data source

The GBD 2019 provides a systematic and comprehensive assessment for 286 causes of death, 369 diseases and injuries, and 87 risk factors globally, covering 204 countries and territories from 1990 to 2019. The data sources of China mainly included censuses, population surveys, Disease Surveillance Points (DSPs) and the Cause of Death Reporting System of the Chinese Centers for Disease Control and Prevention (CDC), and systematic reviews on the incidence and prevalence of various diseases (2). On this basis, GBD used DisMod-MR that allows a systematic analysis of the available demographic and epidemiological data worldwide, ensuring consistency between the morbidity, prevalence and mortality for each disease.

The GBD 2019 estimated the burden of attributable risk factors using a comparative risk assessment (CRA) method. Assuming that the exposure levels of other independent risk factors remain constant, the exposure distribution of a particular risk factor in a specific population is compared with the theoretical minimum risk exposure level (TMREL), and the population attributable fraction (PAF) is calculated (18).

Detailed data sources and up-to-date approaches of data processing have been clearly described elsewhere (2, 18).

In this study, the older people were defined as those aged 60 years and over, based on the legal definition in China. The mortality and DALYs of falls and LBMD-related falls among older people divided by sex in China from 1990 to 2019 were extracted from the Global Health Data Exchange (GHDx), http://ghdx.healthdata.org/gbd-results-tool. The population was divided into five age groups: 60–64 years, 65–69 years, 70–74 years, 75–79 years, and 80+ years. In GBD 2019, falls include death or disability resulting from a sudden movement downward due to slipping, tripping, or other unintentional movement that results in a person coming to rest at a lower level or against an object (2). BMD is measured by dual energy x-ray absorptiometry at the femoral neck (19). LBMD in the GBD study includes both osteopenia and osteoporosis, with osteopenia defined as a T-score between −1 and −2.5, while osteoporosis defined as a T-score equal to or <-2.5 (20).

### Statistical analyses

To estimate the long-term trends for mortality and DALYs rates of LBMD-related falls, the average annual percent changes (AAPCs) and the corresponding 95% confidence intervals (CIs) were evaluated by a joinpoint regression model (version 4.7.0, Joinpoint, IMS, Calverton, MD, USA). The basic idea of this model is to divide a long-term trend line into several segments, each of which can be described as continuous linearity, so as to evaluate the disease change characteristics specific to different intervals time range (21). To quantify the trend over the whole period, AAPC was calculated by weighting the regression coefficients of various annual percent change (APCs). The APC of each segment was calculated using a log-linear model according to APC = [e(β) – 1] × 100%, where β represents the slope of the trend segment (22). A permutation test was used to identify whether the APCs for the corresponding segment were significant, and the best model recommended by the Joinpoint Regression Program was reported. When APC > 0 means that an increase in mortality and DALYs rates of LBMD-related falls was present during this period, and when APC <0 means that a decrease in mortality and DALYs rates of LBMD-related falls was present during this period. Statistical significance defined as the two-sided *p*-value <0.05.

Age-Period-Cohort (APC) analysis is based on the Poisson distribution and can reflect the accumulation of health risks since birth. The general logarithmic linear form of the APC model is (23).


(1) $$\begin{array}{l} \text{ρ}={\text{α}}_{\text{a}}+{\text{β}}_{\text{p}}+{\text{γ}}_{\text{c}} \end{array}$$

where ρ denoted the expected incidence or mortality, and α_a_, β_p_, and γ_c_ denoted the effects of age, period, and cohort of APC model, respectively. Here, the age effect reflects the difference in disease mortality rates due to physiological and pathological changes that occur with age; the period effects reflect difference in changes in disease mortality rates due to various events that occur over time; and the cohort effects reflect differences in disease mortality rates due to lifestyle changes or exposure to risk factors between generations.

To investigate the different effects of age, period and cohort on the mortality trends of falls attributable to LBMD, we used the APC model analysis tool provided by the NCI website (https://analysistools.cancer.gov/apc/) (24). In our model, the age of individuals was restricted to 60+ years and was further divided into 5 successive 5-year groups (from 60 to 64 years group to 80+ years group), while the time period was divided into 6 consecutive 5-year periods (from 1990 to 1994 period to 2015–2019 period). Therefore, 10 corresponding consecutive birth cohorts were generated based on the 5 age groups and 6 period groups: (from 1910 to 1914 group to 1951–1955 group).

The fitted APC model has been used to estimate many useful estimable functions. This study mainly focused on the following estimable functions. The drift (logarithmic linear trend) was used as an estimate of the AAPC of outcome measures over time by period and birth cohort, which incorporated the net drift suggesting the overall percentage change per year and the local net representing the age-specific annual percentage change. The longitudinal age curves were plotted to indicate the age effects by calculating the fitted age-specific rates for the reference cohort when adjusting for period deviations. The effects of the period/cohort were represented by the rate ratios (RRs) relative to the reference period/cohort by controlling for chronological age and the non-linear component of the period/cohort. An absolute drift value of more than 1% was considered as significant change in mortality (25). The Wald chi-squared test was used to determine significance of the estimable functions. A two-sided *p*-value of <0.05 was considered statistically significant.

## Results

**Figure 1:**
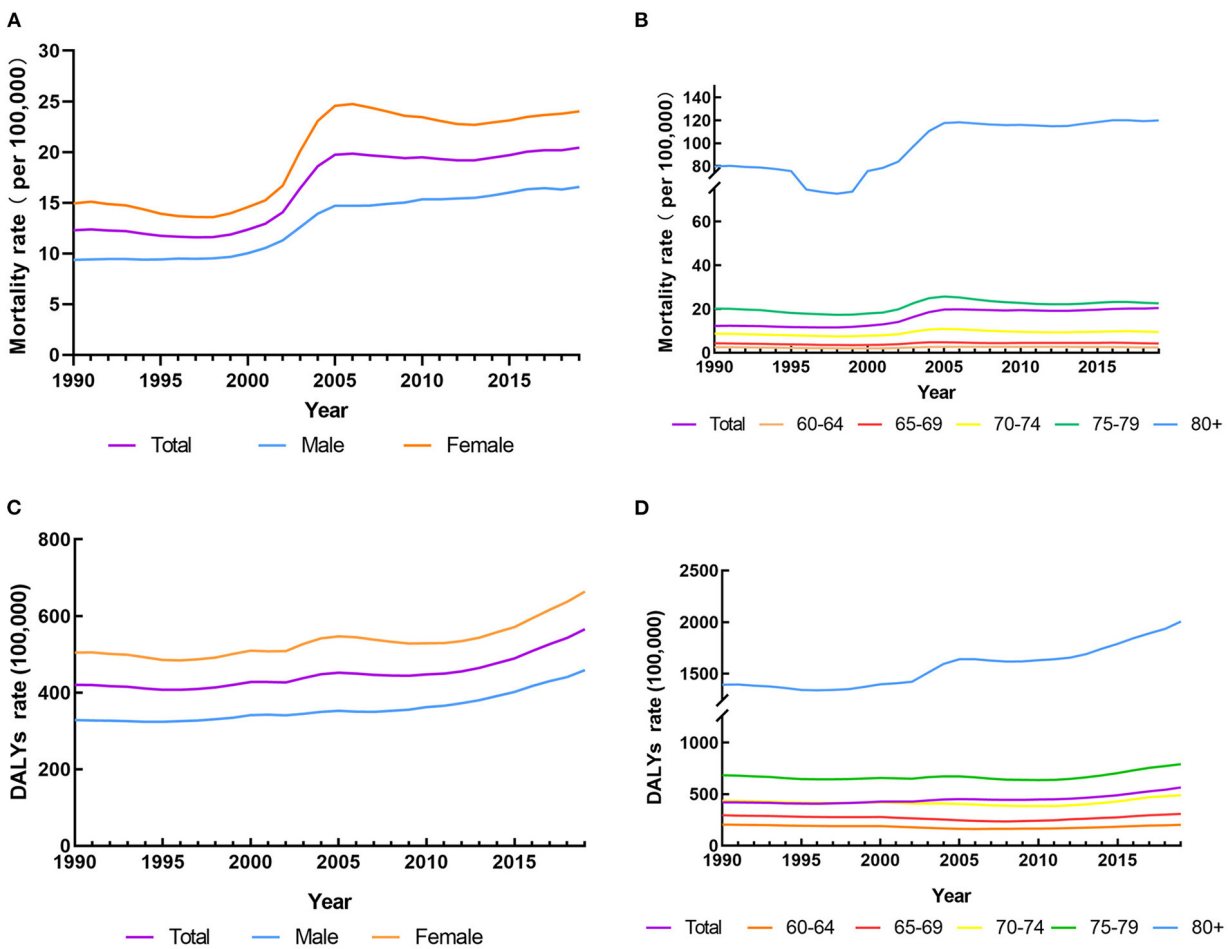
The mortality and DALYs rates of falls attributable to LBMD among people aged 60 years and over in China from 1990 to 2019. **(A)** The mortality rates for both sexes in LBMD. **(B)** The mortality rates for age groups in LBMD. **(C)** The DALYs rates for both sexes in LBMD. **(D)** The DALYs rates for age groups in LBMD.

In 2019, the number of falls deaths among the older people in China was 100,673.50, with approximately half of them (52,513.37 deaths) being attributable to LBMD. The mortality rate, the DALY and the DALY rate caused by falls attributable to LBMD were 20.44 deaths per 100,000 population, 1,451,875.02 person years and 565.14 DALYs per 100,000 population, respectively. From 1990 to 2019, the number of deaths of falls attributable to LBMD among older people in China increased 3.23 times over. The number of deaths in both male and female increased year by year, and the increasing rate of deaths in male was slightly higher than that in female (3.52 times vs. 3.07 times). Fatalities are also on the rise in all age groups, with a growth rate of 4.71 times in people aged 80 years or above (Table 1). Figure 1 shows the temporal trend in the mortality and DALY rates of falls attributable to LBMD among older people in China from 1990 to 2019.

**Table 1:** Mortality, and DALY rates of falls among older people in China in 1990 and 2019, by sex and age groups.

	**1990**				**2019**			
	**Death number**	**Mortality rate, per 100,000 population**	**DALY, person years**	**DALY rate, per 100,000 population**	**Death number**	**Mortality rate, per 100,000 population**	**DALY, person years**	**DALY rate, per 100,000 population**
Total	12,409.93	12.28	424,807.45	420.41	52,513.37	20.44	1,451,875.02	565.14
**Sex group**								
Male	4,514.91	9.367	158,167.93	328.08	20,406.82	16.56	565,144.26	458.53
Female	7,895.02	14.94	266,639.53	504.65	32,106.55	24.02	886,730.76	663.46
**Age group**								
60–64	913.67	2.58	72,633.40	205.06	1,898.39	2.42	158,520.14	201.79
65–69	1,183.73	4.33	80,559.65	294.36	3,020.57	4.29	216,384.43	307.44
70–74	1,644.94	8.72	82,464.93	437.33	4,546.40	9.50	233,855.92	488.67
75–79	2,304.76	20.20	77,918.15	682.86	6,744.03	22.60	236,269.05	791.61
80+	6,362.83	79.62	111,231.34	1,391.90	36,303.98	119.96	606,845.48	2,005.28

DALY, disability-adjusted life-year.

### The temporal trend in the mortality rate and DALY rate of falls attributable to LBMD among people aged 60 years and over from 1990 to 2019

Over the past 30 years, the mortality rate of falls attributable to LBMD among older people in China has increased from 12.28 to 20.44 per 100,000 population with a significant upward trend (AAPC 1.74%, 95% CI, −1.47 to 2.01%), with five identified patterns. Specifically, it slightly decreased from 1990 to 1998 (APC −0.97%) before a sharp increase from 1998 to 2001 (APC 3.87%) and 2001 to 2005 (APC 11.74%), then a downward trend from 2005 to 2012 (APC −0.71%) and a slight increase from 2012 to 2019 (APC 1.00%). Both sexes experienced a significant increase in the mortality rate during the study period (AAPC 1.96% for male and 1.59% for female, respectively). Among age groups, people aged 80 years and older had the largest AAPC (AAPC 1.38%), indicating the largest increase in mortality rate. The AAPCs for the mortality trend in the age of 60–64, 65–69, 70–74, and 75–79 years groups, however, were not statistically significant (Table 2).

**Table 2:** The results by joinpoint regression analysis on the mortality rate of falls attributable to LBMD from 1990 to 2019.

	**Total study period AAPC (%, 95CI)**	**Trend 1**		**Trend 2**		**Trend 3**		**Trend 4**		**Trend 5**		**Trend 6**	
		**Years**	**APC (%)**	**Years**	**APC (%)**	**Years**	**APC (%)**	**Years**	**APC (%)**	**Years**	**APC (%)**	**Years**	**APC (%)**
All	1.74^*^(1.47 to 2.01)	1990–1998	−0.97^*^	1998–2001	3.87^*^	2001–2005	11.74^*^	2005–2012	−0.71^*^	2012–2019	1.00^*^		
**Sex**													
Male	1.96^*^(1.73 to 2.19)	1990–1998	0.07	1998–2001	3.52^*^	2001–2005	8.69^*^	2005–2019	0.87^*^				
Female	1.59^*^(1.23 to 1.95)	1990–1998	−1.53^*^	1998–2001	4.15^*^	2001–2005	13.53^*^	2005–2012	−1.54^*^	2012–2019	0.85^*^		
**Age group**													
60–64	−0.26 (−0.52 to 0.01)	1990–1998	−3.14^*^	1998–2001	0.65	2001–2004	8.97^*^	2004–2009	0.58^*^	2009–2016	−0.57^*^	2016–2019	−2.79^*^
65–69	−0.08 (−0.40 to 0.25)	1990–1998	−2.69^*^	1998–2001	0.91	2001–2004	10.26^*^	2004–2008	−2.05^*^	2008–2016	0.30^*^	2016–2019	−2.16^*^
70–74	0.26 (−0.03 to 0.56)	1990–1998	−2.05^*^	1998–2001	2.35^*^	2001–2005	8.60^*^	2005–2011	−3.02^*^	2011–2017	1.02^*^	2017–2019	−1.73
75–79	0.40 (−0.11 to 0.90)	1990–1998	−2.13^*^	1998–2001	1.69	2001–2004	12.23^*^	2004–2011	−1.93^*^	2011–2019	0.34		
80+	1.38^*^ (1.04 to 1.73)	1990–1998	−1.35^*^	1998–2001	2.16	2001–2005	11.36^*^	2005–2011	−0.65^*^	2011–2019	0.65^*^		

AAPC, the average annual percent change; APC, the annual percent change.

^*^Indicates that AAPC or APC significantly different from 0 (two-sided p < 0.05).

The DALY rate showed an upward trend annually from 1990 to 2019, increasing from 420.41 per 100,000 population to 565.14 per 100,000 population, with an AAPC of 0.99% (95% CI, 0.80–1.19%). The time trend of the DALY rate significantly decreased during 1990–1996 (APC −0.68%) and 2005–2009 (APC −0.38%), but significantly increased during 1996–2005 (APC 1.17%), 2009–2013 (APC 0.99%), and 2013–2019 (APC 3.38%). The DALY rates fluctuated and increased in both sexes (AAPC 1.15% for male and 0.93% for female, respectively) and in the 65–69 (AAPC 0.16%), 70–74 (AAPC 0.39%), 75–79 (AAPC 0.51%), and 80 years and older (AAPC 1.24%) age groups. The AAPC for DALY rate of falls attributable to LBMD in the 60–64 years group, however, was not statistically significant (Table 3).

**Table 3:** The results by joinpoint regression analysis on the DALY rate of falls attributable to LBMD from 1990 to 2019.

	**Total study period AAPC (%, 95CI)**	**Trend 1**		**Trend 2**		**Trend 3**		**Trend 4**		**Trend 5**		**Trend 6**	
		**Years**	**APC (%)**	**Years**	**APC (%)**	**Years**	**APC (%)**	**Years**	**APC (%)**	**Years**	**APC (%)**	**Years**	**APC (%)**
All	0.99^*^ (0.80 to 1.19)	1990–1996	−0.68^*^	1996–2005	1.17^*^	2005–2009	−0.38^*^	2009–2013	0.99^*^	2013–2019	3.38^*^		
**Sex**													
Male	1.15^*^ (0.97 to 1.33)	1990–1995	−0.30	1995–2005	0.85^*^	2005–2008	−0.13	2008–2013	1.55^*^	2013–2019	3.19^*^		
Female	0.93^*^ (0.72 to 1.13)	1990–1996	−0.80^*^	1996–2002	1.02^*^	2002–2005	2.14^*^	2005–2011	−0.75^*^	2011–2015	2.15^*^	2015–2019	3.85^*^
**Age**													
60–64	−0.05 (−0.15 to 0.05)	1990–1996	−1.24^*^	1996–2000	−0.21	2000–2005	−3.12^*^	2005–2011	0.50^*^	2011–2019	2.49^*^		
65–69	0.16^*^ (0.01 to 0.30)	1990–1996	−1.06^*^	1996–2000	0.05	2000–2007	−2.35^*^	2007–2010	0.79	2010–2019	2.82^*^		
70–74	0.39^*^ (0.11 to 0.66)	1990–1997	−0.84^*^	1997–2000	0.72	2000–2010	−1.00^*^	2010–2013	1.45	2013–2017	4.22^*^	2017–2019	2.10^*^
75–79	0.51^*^ (0.38 to 0.64)	1990–1996	−1.11^*^	1996–2005	0.45^*^	2005–2011	−1.00^*^	2011–2019	2.97^*^				
80+	1.24^*^ (1.05 to 1.42)	1990–1997	−0.68^*^	1997–2002	1.41^*^	2002–2005	4.98^*^	2005–2008	−0.84^*^	2008–2012	0.55	2012–2019	2.78^*^

AAPC, the average annual percent change; APC, the annual percent change.

^*^Indicates that AAPC or APC significantly different from 0 (two-sided p < 0.05).

### The age-period-cohort analysis of the mortality rate of falls attributable to LBMD among people aged 60 years and over from 1990 to 2019

**Figure 2:**
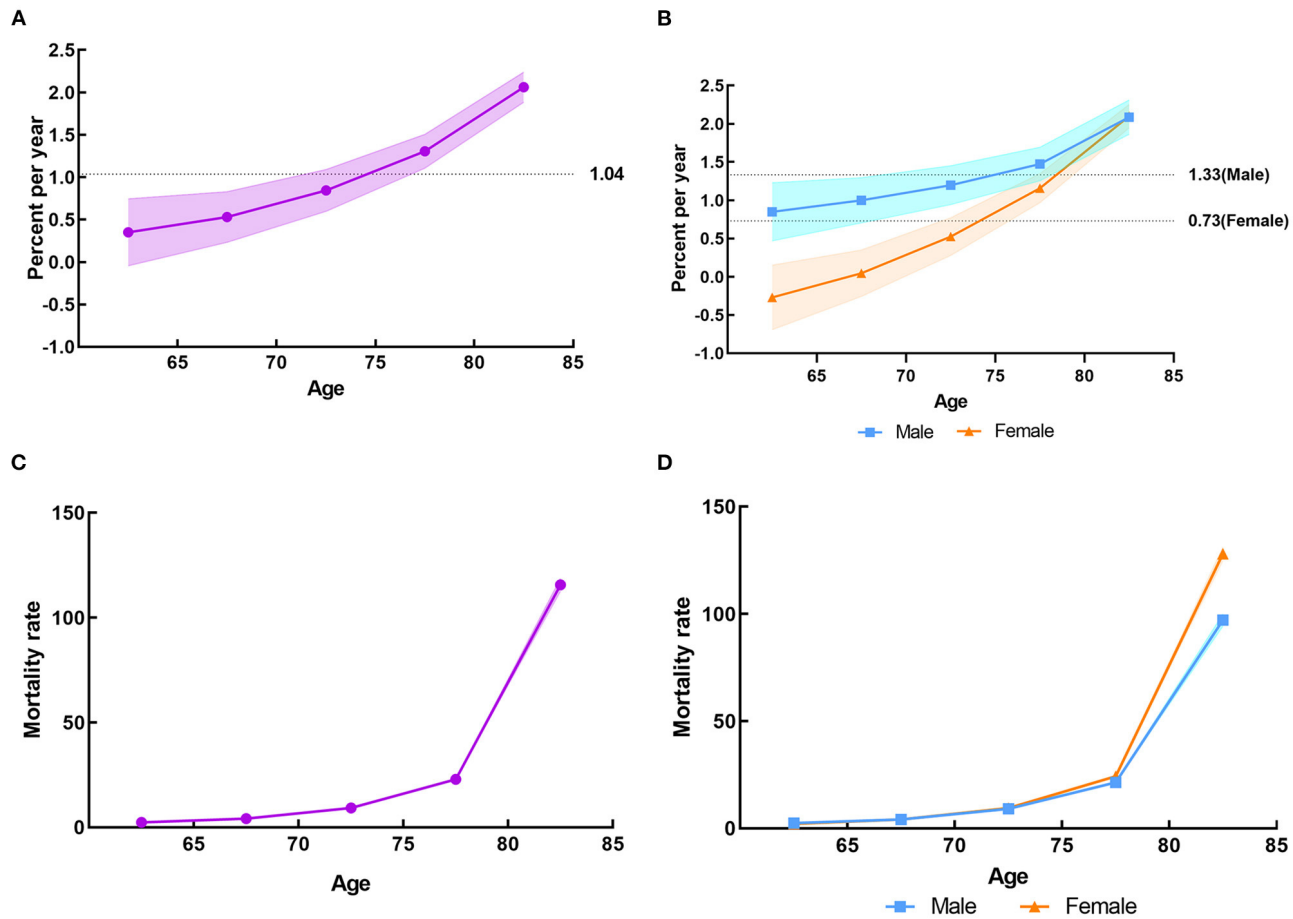
The local drifts and longitudinal age curves of the mortality rate of falls attributable to LBMD among people aged 60 years and over in China from 1990 to 2019. **(A)** The local drifts for both sexes in LBMD. **(B)** The local drifts by sex in LBMD. **(C)** The longitudinal age curves for both sexes in LBMD. **(D)** The longitudinal age curves by sex in LBMD.

Figure 2A shows the overall net drift value per year attributable to LBMD was 1.04% (95% CI, 0.86–1.22%). The local drifts values were above 0 in all age groups, representing an increase in the mortality rates of falls attributable to LBMD. The overall net drifts per year were 1.33% (95% CI, 1.15–1.52%) for male and 0.73% (95% CI, 0.55–0.91%) for female. The local drift values for male and female also increased by age group and were above 0 in the age over 65 years groups (Figure 2B). For the same birth cohort, the mortality attributable to LBMD showed an exponential growth with a rapidly increasing rate at the age of 75 and above group. The mortality among females older than 65 years was higher than that in males (Figures 2C, D).

**Figure 3:**
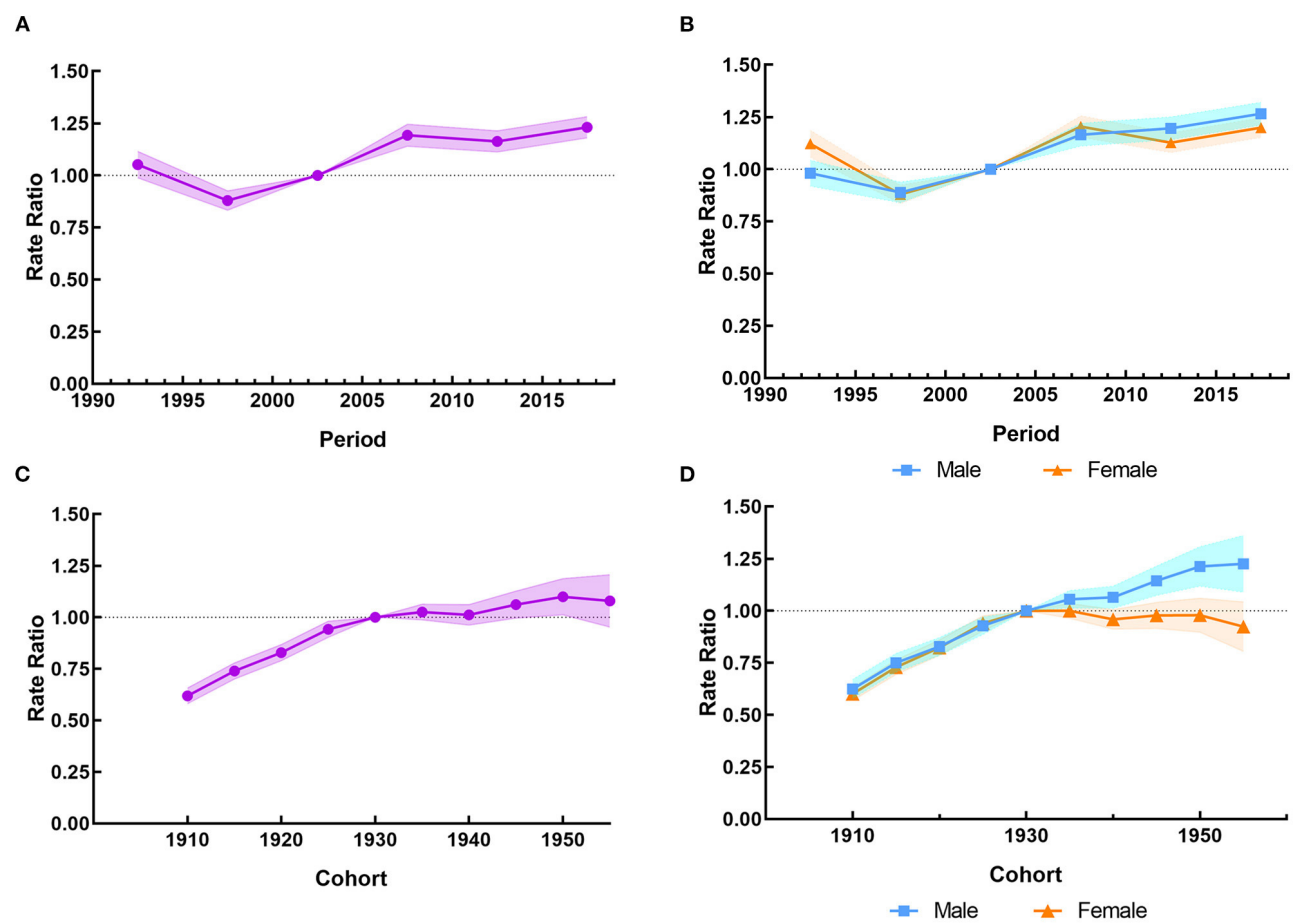
The period RRs and cohort RRs of the mortality rate of falls attributable to LBMD among people aged 60 years and over in China from 1990 to 2019. **(A)** The period RRs for both sexes in LBMD. **(B)** The period RRs by sex in LBMD. **(C)** The cohort RRs for both sexes in LBMD. **(D)** The cohort RRs by sex in LBMD.

Period effects due to LBMD showed a brief decreased trend before 1997, after which the period RRs gradually enhanced, suggesting a progressive increase in the risk of falls mortality for the whole older people groups. It can also be seen that the negative impact of period effects on males was more pronounced than females from 2010 to 2019, with a corresponding RR of more than 1 for both sexes (Figures 3A, B). An overall increasing risk for falls death attributable to LBMD was presented across birth cohorts, but in cohorts born after 1930, the upward trend has slowed down. It was implied that the cohort effect unfavorably affected the mortality of falls attributable to LBMD in the whole older people groups, which was more noticeable in men who were born after 1930 (Figures 3C, D). Figures 2, 3 present the results of the age-period-cohort analysis of the mortality rates of falls attributable to LBMD among older people in China between 1990 and 2019. The age, period and cohort effects were statistically significant, and the detailed results were shown in Supplementary material.

## Discussion

Falls and their consequences are one of the most important causes of injury and disability among the older people. Falls can cause serious injuries such as fracture and craniocerebral injury, resulting in loss of independent living ability or other serious complications or even death. The older population is at a high risk of osteoporosis and falling, which are two closely related and significant health concerns for older adults. Recent studies have shown that osteoporosis increases the risk of falls, which were the most common outcome attributable to LBMD. It has been found that the incidence of falls in Chinese middle-aged and older people with osteoporosis is significantly higher than the average level among community-dwelling older people (26, 27). Therefore, research on the association between LBMD and falls is necessary and important to formulate targeted measures to reduce the disease burden in China.

Using the data derived from GBD 2019, our study is the first to describe the epidemiological trends in mortality and DALYs of falls attributable to LBMD among people aged 60 years and over in China. Several key findings could be drawn from our investigation. Our study revealed that falls attributable to LBMD among people aged 60 years and over showed a significantly increasing trend in mortality and DALYs from 1990 to 2019 in China. Remarkably, the most rapid increase in mortality and DALYs occurred during 1998–2005 and 2009–2019, respectively. It should be noted that the global population is increasing at a high speed (28), and China is no exception. Based on 1990 and 2020 censuses in China, the proportion of people aged 60 years and above has grown from 8.5 to 18.7% over the past 30 years, while the proportion of those aged 80 years and above has grown from 0.7 to 2.5%. It is estimated that by 2050, the population of people aged 60 and older in China will reach 438 million, and China will be in a state of “aging society” for a considerable period of time in the near future. It's possible that as the aging population grows, falls attributable to LBMD may become a more significant burden on the world.

The mortality rates and DALY rates of falls attributable to LBMD in females were higher than those in males. However, the AAPC in the fall mortality rate and DALY rate among men from 1990 to 2019 was higher than that of women, indicating that although the mortality and disability rates of males were lower than those of females, their growth rate was faster. Females are a high-risk and early-onset population for LBMD (29). It has been proven that females have smaller and thinner bone structure than males. After menopause, females are more prone to experiencing bone calcium loss, resulting in LBMD, particularly osteoporosis, due to the lack of protective effects of estrogen on the skeletal system. Epidemiological studies have shown that females suffer more falls than males, and the rate of non-fatal fall injuries is 58% higher than in women that in men (30, 31). This situation is noteworthy. Over the years, attention on LBMD has mainly been focused on women, while the attention to LBMD in men has been consistently underestimated. The overall prevalence of osteoporosis in men over 50 years old is 22.5%, with a fracture incidence of 13%, which is higher than the incidence of prostate cancer (32). Therefore, when formulating fall prevention strategies and measures for the older people with LBMD, consideration should be given to both sexes.

We further analyzed the effects of age, period and cohort on the epidemiological changes in falls attributable to LBMD. In the longitudinal age curves, the substantial increase in the mortality rate of falls attributable to LBMD with aging was observed, it was higher in the age group of 80 years and above. This is related to the characteristics of osteoporosis, an age-related bone disease (33). There are two main reasons: first, an imbalance of bone remodeling caused by aging, manifested as increased bone resorption/bone formation ratio, leading to progressive bone loss; second, the continuous activation of the immune system at a low level and a pro-inflammatory state due to the lack of estrogen (34). Additionally, the oxidative stress and increased glycosylation caused by aging lead to non-enzymatic cross-linking of collagen molecules in the bone matrix, resulting in a decrease in bone strength (35, 36). The older people get, the more likely they are to develop osteoporosis, which will lead to an increase in the severity and risk of death of the older people after falling. Thus, early screening of LBMD and actively prevention falls are of great importance for improving the quality of life in old age.

The results also showed that the RRs of LBMD- related falls mortality rates in China changed in the 1997s, with higher RRs observed after 2002. The increased RR period after the 1997s was largely due to the dilemmas in diagnosis and treatment of osteoporosis in China. As early as the 1990s, China has strengthened the prevention and treatment against osteoporosis. For example, since 1994, the prevention and treatment of osteoporosis has been included in the national support projects of the Ninth Five-Year Plan, the Tenth Five-Year Plan, and the Eleventh Five-Year Plan in China. In 2003, the former Ministry of Health approved the introduction of an international training course on bone mineral density measurement to improve the diagnosis capabilities of osteoporosis in China (32). Unfortunately, due to the uneven distribution of medical resources, the diagnosis rate is still low in some under-developed rural areas. Wang reported that BMD measurement had never been performed on more than 60% of the 1993 patients before or after the fragility fractures in China (37). Insufficient diagnosis of the disease may directly result in inadequate use of anti-osteoporotic medication. Yu reported that in a 30% random selection of the Tianjin Urban Employee Basic Medical Insurance (UEBMI) database between 2008 and 2011, 69,476 individuals were diagnosed with osteoporosis, and only 20.6% of them received medication (38). A retrospective cohort study was carried out based on an existing electronic health record database. And the results showed that the usage rate of anti-osteoporotic drug therapy after osteoporotic fractures in China is low (22.1% for women and 9.5% for men) (39). Besides, the treatment rate of anti-osteoporosis medication (AOM) had a slight downward trend during 2010–2016. As a result, osteoporosis might be under-treated in China.

The RR of cohort effects on falls mortality due to LBMD was on the rise. As we have mentioned, LBMD is an age-related condition mainly affecting older people, thus an extension of life expectancy and an increase in proportion of older people may have a role. On the other hand, with the development of socio-economy and the improvement of living standards, urbanization of lifestyle among older people in China is becoming more common, including living in higher floors, sedentary lifestyle for longer time, less exposure to sunlight, less physical activity and a more Westernized diet, which has led to the increase of LBMD incidence (40). In addition, due to the increase of empty nesters, the delay in medical care and quality after a fall, and the lack of care will also cause serious consequences of disability or death (41).

The pattern of increase in cohort RRs has slowed since the 1930s among older adults, which may be related to the increased availability of drugs and effective treatment for osteoporosis. Moreover, the publication of “Guidelines for osteoporosis and Bone mineral disease” in 2006 and “Guideline for primary osteoporosis” in 2011 might have resulted in a positive impact on increasing awareness of osteoporosis in China. Younger birth cohorts have received education on the subject earlier, leading to better treatment adherence. It should be noted that the use of anti-osteoporotic drug was less common in male patients than in female patients in almost all the studies available. It might associate with weaker awareness of osteoporosis in men and the hidden progression of the disease.

During the past decades, although prevalence of LBMD declined worldwide, the absolute death number and DALYs attributable to LBMD increased in almost all countries. China has both the largest older population and the largest rate of increase of older people in the world. Considering such a large population of seniors, it is very important to explore factors like LBMD including osteoporosis, which may cause falls and deteriorate older people's health. Studies indicated that in some countries, such as the United States, Australia, Canada, and China, the disability and mortality rates of LBMD increase over time (26). It can be seen that once older people with osteoporosis fall, it will definitely increase the medical and economic burden on society and families. It will make the management more challenging if efforts are not fully taken to deal with LBMD and preventable falls in older population.

The prevention and treatment system of LBMD can be divided into three levels: “Disease prevention,” “Complication prevention,” and “Disability prevention”. Disease prevention is to prevent the occurrence of LBMD, mainly by practicing a bone-healthy lifestyle. It has been proven that a lack of nutrient intake, such as such as high-quality protein, calcium, and vitamin D, predict lower bone mass (42). It is recommended to increase bone reserve before age of 30 to ensure higher bone mass in their future life and reduce bone loss after age of 50. It is advisable to increase the time and area of sun exposure to facilitate the synthesis of vitamin D in the body. At the same time, regular physical activity and exercise can also help improve strengthen muscles, improve body flexibility and enhance reactive responses to reduce the risk of falls (43, 44). In fact, diagnosis and treatment of osteoporosis in male has been overlooked for a long time, which may explain lower mortality and disability but higher growth rate in male than in female in this study. Complication prevention means early screening, standard anti-osteoporosis drug treatment, fall and fracture prevention on the basis of primary prevention. One of the main objectives of early diagnosis is to develop useful osteoporosis assessment tools to identify people at high risk, and enable them to benefit from early intervention. The use of calcium and/or vitamin D supplements in combination with anti-osteoporosis drugs is recommended for older patients with osteoporosis to reduce the risk of fractures and falls (45). Therefore, it is of great importance for clinicians to gain awareness of detection and treatment of osteoporosis in men. Moreover, treatment plans for osteoporosis in older people should be gender-equal.

Falls are often linked to hip fractures, one type of the two typical osteoporotic fractures (46). Conversely, hip fractures can be caused by the mechanical forces on falling, leading to substantial disability and increased risk of mortality. Disability prevention refers to implement active interventions, such as surgery for patients with fracture or at high risk of fracture, to maintain the patients' quality of life as much as possible and reduce disability and early death. Therefore, the establishment of a tertiary prevention and control system is of great significance for improving the prevention and treatment level of osteoporosis in the older people, reducing the disease burden of falls in the older people, and achieving a society of healthy aging.

## Limitation

There are still several limitations in this study. Firstly, this study only described and analyzed GBD 2019 datasets within China, without further evaluating the differences among provinces, and between urban and rural areas. Secondly, the change in the burden of falls might be affected by the shift in the population age structure. In this study, data for those aged 60 and above are not age-standardized. Finally, the GBD study was conducted by using system dynamics modeling and statistical modeling to derive estimates from limited raw data, which resulted in unavoidable result distortion due to heavy assumptions.

Nevertheless, there's something new in this study. This is the first study to analyze the age-period-cohort effects in the temporal trends in falls mortality attributable to LBMD, suggesting that LBMD is related to falls. In China, developing plans for preventing LBMD may be a potentially cost-effective strategy to reduce the burden of fractures and falls, particularly in the aging population who are increasingly affected.

## Conclusion

After adjusting for period deviations, falls mortality rate due to LBMD has been increasing from 1990 to 2019, particularly in the older people aged 80 and older. Among older men born in the post-1930s period, this situation has been gradually worsening, with a negative period effect observed from 2010 to 2019 when compared to women. The treatment of osteoporosis has been neglected in men for a long time. With the upcoming aging population in China in the near future, we will face increasing challenges. The prevalence of chronic diseases is expected to increase, which has been shown to lead to an increased risk of severe falls and death among older adults (47). Additional efforts and resources are necessary from national and local governments to establish a solid support system, such as health promotion strategies, policies, and interventions, to alleviate and control the severe situation of LBMD, thus reducing the impact of LBMD on burden of falls.

## Data availability statement

Publicly available datasets were analyzed in this study. This data can be found here: https://vizhub.healthdata.org/gbd-results/.

## Author contributions

YF and LB designed the study and conceived analysis plan. YF collected and analyzed data and wrote the first draft of the manuscript. HY and XH directed statistical analyses of the data and revised the paper. NL, JZ, and ZS reviewed and revised the manuscript. All authors have read and approved the submitted version of the manuscript.
