# Supplementary material for: Burden of falls attributable to low bone mineral density among people aged 60 years and over in China from 1990 to 2019
Source: Front Public Health. 2023 Jun 28;11:1204497. doi: 10.3389/fpubh.2023.1204497 (PMC10338097; doi:10.3389/fpubh.2023.1204497)
Supplement: Supplementary file 1 [file Data_Sheet_1.docx]

**Supplementary materials**

**The steps in searching data on the online GBD tool**

1. Go to GBD database ( the website: <http://ghdx.healthdata.org/gbd-results-tool>).
2. Specify our query parameters using the tool’s controls. The figure was the GBD Results Tool user interface.


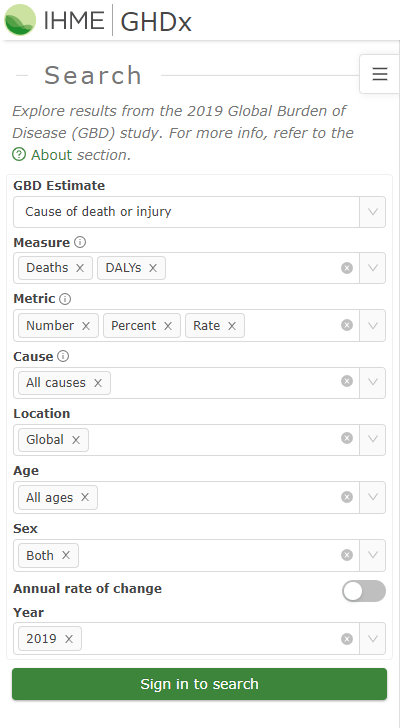


In our studies, the measure was defined as deaths and DALYs, location was defined as China; Age was defined as 60-64 age-group, 65-69 age-group, 70-74 age-group, 75-79 age-group, and 80 plus age-group; Sex was defined as both, male and female. Year was defined as 1990 to 2019. Metric was defined as number and rate. Cause was defined as falls. In GBD 2019, falls were defined as a sudden movement downwards due to slipping, tripping, or other unintentional motion that results in a person coming to rest at a lower level or against an object. Risk was defined as low bone mineral density. LBMD in the GBD study includes both osteopenia and osteoporosis.

1. After all spaces are selected, and download relevant data.
